# Correction: All-solution-processed, flexible thin-film transistor based on PANI/PETA as gate/gate insulator

**DOI:** 10.1039/c8ra90105h

**Published:** 2019-01-29

**Authors:** Jin-Yong Hong, Kyoung-Hwan Shin, Dai Gun Yoon, Byung Doo Chin, Sung Hyun Kim

**Affiliations:** Department of Electrical Engineering and Computer Science, Massachusetts Institute of Technology 77 Massachusetts Avenue Cambridge MA 02139 USA; School of Chemical and Biological Engineering, Seoul National University Seoul 151-747 Republic of Korea; Department of Polymer Science and Engineering, Dankook University Youngin 448-701 Republic of Korea bdchin@dankook.ac.kr; Department of Chemistry, College of Natural Sciences, Seoul National University Seoul 151-747 Republic of Korea shkim75@snu.ac.kr

## Abstract

Correction for ‘All-solution-processed, flexible thin-film transistor based on PANI/PETA as gate/gate insulator’ by Jin-Yong Hong *et al.*, *RSC Adv.*, 2015, **5**, 105785–105788.

The authors regret the omission of one of the authors, Kyoung-Hwan Shin, from the original manuscript. The corrected author list is as shown above.

The Royal Society of Chemistry apologises for these errors and any consequent inconvenience to authors and readers.

## Supplementary Material

